# Admission selection criteria as predictors of outcomes in an undergraduate medical course: A prospective study

**DOI:** 10.3109/0142159X.2011.577123

**Published:** 2011-05-19

**Authors:** Annette Mercer, Ian B Puddey

**Affiliations:** The University of Western Australia, Australia

## Abstract

**Background:**

In 1998, a new selection process which utilised an aptitude test and an interview in addition to previous academic achievement was introduced into an Australian undergraduate medical course.

**Aims:**

To test the outcomes of the selection criteria over an 11-year period.

**Methods:**

1174 students who entered the course from secondary school and who enrolled in the MBBS from 1999 through 2009 were studied in relation to specific course outcomes. Regression analyses using entry scores, sex and age as independent variables were tested for their relative value in predicting subsequent academic performance in the 6-year course. The main outcome measures were assessed by weighted average mark for each academic year level; together with results in specific units, defined as either ‘knowledge'-based or ‘clinically’ based.

**Results:**

Previous academic performance and female sex were the major independent positive predictors of performance in the course. The interview score showed positive predictive power during the latter years of the course and in a range of ‘clinically' based units. This relationship was mediated predominantly by the score for communication skills.

**Conclusions:**

Results support combining prior academic achievement with the assessment of communication skills in a structured interview as selection criteria into this undergraduate medical course.

## Introduction

Methods of selection of students for entry to medical courses have changed in recent years to include components other than previous academic achievement (Mercer 2009). The inclusion of alternative components of selection such as aptitude tests and some form of interview has been controversial (Powis 2008) and the paradigm shift away from the exclusive use of academic scores has been slow (Edwards et al. 2001). In Australia, the use of the three components: academic score, selection interview and the Undergraduate Medicine and Health Sciences Admission Test (UMAT), has been common among the undergraduate medical schools since the late 1990s. These three components are used in quite different ways in the selection processes of the various universities and each university has developed its own form of interview (Mercer 2009).

The significant increase in demand for a medical education has contributed to shaping new methods of selection at both the graduate and undergraduate levels (Elliott & Epstein 2005). A major issue in these alternative methods is the determination of a valid, reliable, fair and transparent method of distinguishing between the many applicants who are suitably academically qualified to enter a medical course. One of the major reasons for the proliferation of intellectual aptitude tests (McManus et al. 2005) in the UK is the difficulty in distinguishing between the growing numbers of applicants achieving three A grades at A-level. A similar situation exists in Australia (Story & Mercer 2005) with a large number of medical school applicants achieving a high Tertiary Entrance Rank (TER, Table 1). The Australian Council of Educational Research, the developers of UMAT, specify that it is designed to assess general attributes and abilities gained through prior experience and learning; specifically, the acquisition of skills in critical thinking and problem solving, understanding people and abstract non-verbal reasoning. These abilities are considered important to the study and later practice of professions in the health sciences (Mercer & Chiavaroli 2006). Each of these attributes is operationalised as a cognitive skill, hence UMAT assesses skills different from those assessed in the interview.

**Table 1:** Selection into the 6-year undergraduate medical course at the UWA.

Standard (school-leaver) applicants	
1. TER	Minimum rank of 96 on a scale to 99.95
An academic score formed from the aggregate of results in the state Tertiary Entrance Examinations held at the end of secondary school	
2. UMAT total score of three sections:	Threshold set each year.
UMAT_1 Logical reasoning and problem solving	Each section is standardised to a mean of 50 and standard deviation of 10
UMAT_2 Understanding people	
UMAT_3 Non-verbal reasoning	
3. Score on structured interview (Table 2)	Threshold set each year
Six criteria plus a global score for communication skills	1999-2005, maximum score of 28
The six criteria were varied each year for security purposes	2006-2009, maximum score of 42 (revised scale)

*Note:* Ranking of applicants is by a combined score using the three components. Initially the three components were weighted equally. From 2007 entry, they were weighted in the ratio 2:2:1 for TER, Interview score, UMAT.

**Practice points**

- A structured interview emphasising communication skills can add value to the selection of school-leavers into a medical course.
- The interview score was most closely associated with clinical outcomes.
- Previous academic achievement and female sex were consistent predictors of course outcomes.
- The effects of interview scores and aptitude test scores should continue to be evaluated post-graduation.

Furthermore, an understanding of the characteristics of a good doctor is evolving with general agreement from most quarters that both interpersonal and cognitive characteristics are important qualities for doctors to possess (Fones et al. 1998; McGaghie 2002; Cullen et al. 2003; Powis 2008). Foremost amongst these characteristics is the ability to communicate with peers and patients, and the selection interview has developed in its many forms in an attempt to assess such qualities (Powis 2008; Mercer 2009). The use of the modern structured or semi-structured interview has a relatively short and controversial history in this context (Mercer 2009). It is costly to administer and results on its predictive validity for student performance have been inconsistent (Hughes 2002). This has led to at least one graduate medical school in Australia abandoning its use and relying on aptitude tests and academic performance alone (Wilkinson et al. 2008).

Good communication skills are seen as important attributes for both medical students and doctors. Modern medical curricula generally include units on the development of these skills, in spite of complaints from students about time spent on such courses (Rees et al. 2003). An Australian study (Hyde et al. 2010) which surveyed doctors recently registered to practise found that when asked which medical course areas helped them most in accessing further training, they put the area of Communication Skills training first. The authors concluded that the personal qualities of doctors were considered more influential in accessing further training than the features of a medical course. Hence, they suggested that more emphasis should be put on selecting candidates with the required attributes and they noted the implications for medical schools’ admissions criteria.

In 1998, the Faculty of Medicine and Dentistry at The University of Western Australia (UWA) introduced a new form of admission to its 6-year undergraduate MBBS course. Details can be seen in Table 1. This study reports on Standard entrants who have just completed secondary school and who comprise more than 80% of students in the course. Non-standard (some tertiary study) entry students who may have completed as little as 1 year of tertiary study have been similarly studied, but the results will not be reported in detail here, mainly due to the considerably smaller numbers involved (249 over the 11-year period) and the different academic scores used for entry (Grade Point Average, GPA). The faculty also conducts a graduate entry programme which was not included in this study.

The aim of this study was to determine the relationship between the combination of Standard (school-leaver) medical students’ entry scores and some demographic characteristics and subsequent student performance in the undergraduate course. The role of the interview score was a particular focus in the study. The study was approved by the university's Human Research Ethics Committee.

## Methods

### The participants

The first 11 cohorts of students selected using this new process, that enrolled from 1999 through 2009, were followed serially in relation to specific course outcomes at the end of 2009. The majority of those enrolled from 1999 through 2004 had graduated from the 6-year undergraduate course, the majority from 2005 were in their final year and the remainder of entrants were progressing through the course, with the majority of those who commenced in 2009 having completed their first year. Hence, the quantity of data for each academic year of the course varies from 1174 in Year 1 to 547 in Year 6 for Standard entrants. All entrants via this selection process were studied, including those who withdrew or were excluded for unsatisfactory progress. In cases where students had repeated a unit their first unit score was included in the analysis. International full fee paying students and indigenous students admitted via special entry criteria were not included in the study.

### Predictor variables

In addition to the academic score (TER), predictor variables included the total interview score and the UMAT score. Even though the total UMAT score was used in the ranking process, the three component scores UMAT_1, UMAT_2 and UMAT_3 were used in this study because of the different and independent constructs underlying the three sections (Mercer & Chiavaroli 2006). The structured interview process utilised is outlined in Table 2. The total interview score was used as the predictor variable rather than individual criterion scores because the interview questions and the criteria assessed varied across each year for reasons of interview security. A global score for communication skills was a consistent component of the total interview score each year and hence separate analyses were also able to be conducted with models that utilised the *communication skills* score instead of the total interview score.

**Table 2:** The structured interview.

• Interviews were conducted by a panel of two consisting of a male and a female, a university member and a community member; and all interviewers were required to re-train each year
• Six criteria were assessed each year using three set questions for each criterion. The seventh criterion *communication skills* was assessed across the responses to the set questions
• The interview had a highly structured format in which all applicants were asked exactly the same questions and only standard prompts were used
• The basic format of the interview remained consistent over the years, with changes to the rating scales in 2006. Originally, the seven criteria were each scored 0-4, more recently each criterion was scored 0-6
• The final score was a consensus score determined after each interviewer had assessed the responses independently against clearly defined rating scales
• A bank of criteria had been developed. The criteria were based on qualities suited to the study and practice of medicine, such as *ability to work in a team, ability to see from the perspective of others, social responsibility, recognising and responding to social diversity, ethics, coping with uncertainty, etc*. After the criteria were selected each year, the questions and rating scales were revised or developed by a committee of five. One new criterion was developed each year
• The criteria *commitment and motivation to study medicine* and *communication skills* were assessed each year
• The assessment of *communication skills* was across four domains: comprehension, articulation, relevancy and interaction
• The time allocation for an interview was 60-70 min, with the actual interview averaging 35 min and the remainder of the time being used for individual and consensus ratings

To overcome variations in the distribution of UMAT scores and interview scores over the 11 cohorts, standardised scores (Z-scores with a mean of 0 and standard deviation of 1) were calculated within each cohort for the three sections of UMAT and the interview total score. In addition to these scores, sex (female = 0, male = 1) and age were included in each regression model. Hence the final set of predictor variables consisted of TER, age, sex, and Z-scores for UMAT_1, UMAT_2, UMAT_3 and the interview total score (or *communication skills* score).

### Outcome measures

The individual academic year Weighted Average Mark (WAM) was calculated for all core units for Year 1 through Year 6. In each case, the analyses include all units completed by the end of 2009. The score for each unit is ‘weighted’ according to the size and hence relative importance of the unit. Secondary outcome variables included the mark for a range of individual units which were selected to assess the relative contribution to the WAM of performance in specific units that were either ‘knowledge'-based or ‘clinically’ based. For the former, the curriculum was delivered mainly in didactic fashion in lectures and laboratory sessions; and assessment was predominantly of factual knowledge. For the latter, the curriculum was delivered through a combination of problem-based learning tutorials, case-based tutorials or clinical teaching; and assessment was either through a multidisciplinary observed structured clinical examination or a composite assessment of clinical performance. In all cases unit results were recorded as percentages, rather than pass/fail or grades.

### Statistical analysis

Data were analysed in SPSS for Windows version 15.0. Summary statistics at entry were compared across each cohort (1999-2009) by either one-way ANOVA for continuous variables or chi-squared statistic for categorical variables. Pearson correlation coefficients were calculated for the Year level WAM (Years 1-6) with each of the predictor variables listed above.

Linear regression models were constructed for each outcome variable using the full set of predictor variables. The estimates obtained from the linear regression models are reported without correction. Two forms of correction may be applied in studies such as this. One is the Bonferroni correction for multiple comparisons (Wilkinson et al. 2008) which is applied *post-hoc* by a reduction in the nominal level of significance for the combined test. The other is a correction for restriction of range of the criterion variables, which usually results in higher correlations in predictive validity studies (Wiberg & Sunderstrom 2009). The standard deviations of the population scores are not known, hence this correction has not been applied.

## Results

### Student summary statistics

The recorded demographics, sex and age, were not significantly different across cohorts. The mean age across cohorts was approximately 18 years and the proportion of females to males was 56% (F) to 44% (M). Some small, but statistically significant, correlations existed between the TER and each of the three sections of UMAT (UMAT_1: *r*= 0.137, *p* < 0.001; UMAT_2: *r* = -0.078, *p* < 0.01; and UMAT_3: *r*= 0.216, *p* < 0.001, respectively). However, these inter-correlations were considered small enough to not unduly influence the regression models.

### Correlation coefficients

Pearson correlation coefficients of the predictor variables against the Year level WAM for core units throughout the course are presented in Table 3. This table shows that TER has the highest correlation with the WAM in Years 1-3, whereas TER and female sex are approximately equal in magnitude in Years *4—6 (p <* 0.001). The magnitude of the correlation with sex is fairly consistent over the six Year levels but slightly higher in Year 5. The interview score becomes relevant in Years 4-6 with *p* < 0.01. Year 5 WAM has a significant correlation at the 5% level with UMAT_1 (positive) and UMAT_3 (negative). Statistically significant results are shown in bold in Tables 3-6.

**Table 3:** Pearson correlation coefficients for each predictor variable versus the year level WAM.

		WAM Year 1 (*N* = 1174)	WAM Year 2 (*N* = 1017)	WAM Year 3 (*N* = 901)	WAM Year 4 (*N* = 799)	WAM Year 5 (*N* = 688)	WAM Year 6 (*N* = 547)
Age at entry	Pearson correlation	-0.018	-0.036	-0.031	-0.065	-0.044	-0.038
	Significance (2-tailed)	0.536	0.245	0.350	0.066	0.253	0.375
Sex	Pearson correlation	-0.112	-0.122	-0.143	-0.226	-0.308	-0.210
	Significance (2-tailed)	< **0.001**	< **0.001**	< **0.001**	< **0.001**	< **0.001**	< **0.001**
TER	Pearson correlation	0.468	0.401	0.321	0.230	0.208	0.206
	Significance (2-tailed)	< **0.001**	< **0.001**	< **0.001**	< **0.001**	< **0.001**	< **0.001**
Z-score: interview score	Pearson correlation	0.012	0.040	0.054	0.138	0.102	0.111
	Significance (2-tailed)	0.675	0.202	0.104	< **0.001**	**0.007**	**0.009**
Z-score: UMAT total score	Pearson correlation	0.030	-0.001	-0.006	0.035	0.038	-0.044
	Significance (2-tailed)	0.308	0.970	0.860	0.316	0.318	0.299
Z-score: UMAT1 - logical reasoning and problem solving	Pearson correlation	0.043	0.011	0.005	0.057	0.085	0.042
	Significance (2-tailed)	0.136	0.726	0.892	0.107	**0.026**	0.322
Z-score: UMAT2 - interaction skills	Pearson correlation	-0.014	-0.044	-0.009	0.039	0.056	-0.023
	Significance (2-tailed)	0.634	0.160	0.787	0.276	0.143	0.595
Z-score: UMAT3 - non-verbal reasoning	Pearson correlation	0.034	-0.003	0.009	-0.045	-0.086	-0.070
	Significance (2-tailed)	0.243	0.929	0.795	0.203	**0.025**	0.104

**Table 4:** Regression models of the relationship between selection criteria for standard entry students 1999–2009 and academic performance as assessed by yearly WAM.

	WAM Year 1 (*N* = 1174)	WAM Year 2 (*N* = 1017)	WAM Year 3 (*N* = 901)	WAM Year 4 (*N* = 799)	WAM Year 5 (*N* = 688)	WAM Year 6 (*N* = 547)
Age (year)						
Beta	0.059	0.027	0.021	−0.026	−0.011	−0.001
*p*-value	**0.022**	0.340	0.513	0.430	0.625	0.975
Sex (F=0/M=1)						
Beta	−0.151	−0.16	−0.176	−0.245	−0.328	−0.235
*p*-value	**<0.001**	**<0.001**	**<0.001**	**<0.001**	**<0.001**	**<0.001**
TER score						
Beta	0.505	0.442	0.359	0.272	0.257	0.246
p-value	**<0.001**	**<0.001**	**<0.001**	**<0.001**	**<0.001**	**<0.001**
Interview Z-score						
Beta	0.027	0.046	0.061	0.139	0.089	0.114
*p*-value	0.306	0.207	0.054	**<0.001**	**0.012**	**0.006**
UMAT 1 Z-score						
Beta	−0.005	−0.031	−0.023	0.048	0.068	0.030
*p*-value	0.837	0.165	0.473	0.56	0.058	0.467
UMAT 2 Z-score						
Beta	−0.015	−0.047	−0.013	0.016	0.018	−0.038
*p*-value	0.571	0.092	0.682	0.629	0.614	0.369
UMAT 3 Z-score						
Beta	−0.047	−0.072	−0.027	−0.025	−0.048	−0.037
*p*-value	0.076	0.071	0.405	0.462	0.187	0.389
R^2^ (%)	**25.1**	**20.0**	**14.3**	**14.3**	**17.5**	**11.7**

**Table 5:** Regression models of the relationship between selection criteria and academic performance in specific ‘knowledge’-based units.

	Normal systems (levels 1 and 2) (*N* = 1074)	Pathology, pharmacology, microbiology (level 3) (*N* = 901)	Science and practice of medicine (levels 4-6) (*N* =799)
Age (year)			
Beta	-0.012	0.023	-0.022
*p*-value	0.676	0.467	0.511
Sex(F = 0/M = 1)			
Beta	-0.087	-0.131	-0.227
*p*-value	**0.002**	< **0.001**	< **0.001**
TER			
Beta	0.462	0.360	0.227
*p*-value	**0.001**	< **0.001**	**0.001**
Interview Z-score			
Beta	0.031	0.042	0.084
*p*-value	0.259	0.183	**0.014**
UMAT 1 Z-score			
Beta	-0.040	-0.029	0.096
*p*-value	0.151	0.359	**0.005**
UMAT 2 Z-score			
Beta	-0.043	-0.011	-0.008
*p*-value	0.130	0.729	0.814
UMAT 3 Z-score			
Beta	-0.043	-0.036	-0.056
*p*-value	0.130	0.265	0.109
*R^2^* (%)	**20.3**	**13.1**	**11.4**

**Table 6:** Regression models of the relationship between selection criteria and academic performance in specific ‘clinically’ based units.

	Foundations of clinical practice (levels 1–3) (N=1165)	Clinical skills (levels 4–6) (N=799)	General practice (levels 5 nd 6) (N=688)
Age (year)			
Beta	0.023	−0.024	−0.050
*p*-value	0.384	0.478	0.176
Sex (F=0/M=1)			
Beta	−0.336	−0.171	−0.255
*p*-value	<**0.001**	<**0.001**	<**0.001**
TER			
Beta	0.352	0.182	0.132
*p*-value	<**0.001**	<**0.001**	0.001
Interview Z-score			
Beta	0.075	0.182	0.131
*p*-value	**0.005**	<**0.001**	<**0.001**
UMAT 1 Z-score			
Beta	−0.049	0.077	0.050
*p*-value	0.064	**0.026**	0.180
UMAT 2 Z-score			
Beta	−0.007	−0.028	0.011
*p*-value	0.784	0.418	0.762
UMAT 3 Z-score			
Beta	−0.078	−0.061	0.008
*p*-value	**0.004**	0.084	0.828
R2 (%)	**22.6**	**10.1**	**10.0**

### The regression analyses

*Yearly WAM*. Table 4 shows that the amount of variance in the yearly WAM for the core units accounted for by the independent variables ranged from 25% in Year 1 to approximately half that amount by Year 6. The strongest predictors of a higher WAM in each academic year were TER and female sex *(p <* 0.001). The significance of the beta coefficient associated with TER diminished from Year 1 to Year 6 while the sex effect remained relatively consistent across each year. The other substantive predictor of a higher WAM was the standardised interview score, which became a significant predictor in Years *4—6 (p <* 0.01 for Years 4 and 6 and marginal in Year 5).

*Knowledge-based units*. Table 5 shows an illustrative selection of the analysis of ‘knowledge'-based units across Years 1-6. The TER and female sex were the consistent predictors of a higher mark. A significant influence from the interview score was seen in the Science and Practice of Medicine unit *(p <* 0.05), spanning Years *4—6*. UMAT_1 (logical reasoning and problem solving) was also a significant predictor for the score in Science and Practice of Medicine *(p <* 0.01).

*Clinically based units*. Table 6 shows an illustrative selection of the analysis of ‘clinically’ based units across Years 1-6. TER and female sex were the consistent predictors of a higher mark. In contrast to the ‘knowledge'-based units, the interview score also predicted a higher mark in ‘clinically’ based units at all levels of the course. UMAT_1 (logical reasoning and problem solving) was a significant positive predictor for a higher mark in the Clinical Skills unit (Years *4—6)* while UMAT_3 (non-verbal reasoning) was a significant negative predictor for Foundations of Clinical Practice (Years 1-3).

*Global communication score*. All analyses were repeated replacing the total interview score with the single score for *communication skills*. The outcomes from the two sets of analyses were almost identical.

## Discussion

Selection into the undergraduate course at the medical school at UWA is based on a composite entry score derived from prior academic performance, a structured selection interview and attributes and abilities determined by the UMAT. The aim of this study was to determine the relative utility of each of the individual components of the entry score as independent predictors of medical students’ subsequent performance during the course, with a focus on the interview score. This focus is generated by two factors: the significant cost of the interview, especially in terms of the human resources invested in it, as well as the uniqueness of this particular interview to our setting.

Previous academic achievement (TER) and female sex were consistent predictors of better performance, with the effect of TER diminishing over the Year levels and the sex effect remaining at a consistent level. The interview score proved a significant positive predictor, with this effect seen particularly in the clinical years but also evident for individual illustrative clinical units at all year levels. Sections of the UMAT produced inconsistent results, with UMAT_1 (logical reasoning and problem-solving) being the most consistent. Each predictor variable will be discussed separately.

### Academic score

Prior academic achievement was the predominant and most consistent independent predictor of success in our MBBS course. The effect of TER was highest in the early academic years and diminished towards the end of the course. Such an effect of previous academic achievement is consistent with previous research findings both in medical courses (Ferguson et al. 2002; Hughes 2002) and for tertiary study in general (Dobson & Skuja 2005; Win & Miller 2005; Birch & Miller 2007). In a long-term study of medical graduates in the UK, McManus et al. (2003) concluded that previous academic achievement (as measured by A-Level results) not only predicted outcomes in a medical course but also those during subsequent careers. The results of this study simply confirm the place of this component in the selection algorithm.

### Female sex

An effect of sex has not always been considered by previous researchers in higher education studies (Win & Miller 2005). In our students, we have demonstrated that females consistently performed better than males, an effect seen throughout the course. This supports findings by Ferguson et al. (2002) and suggests that this variable should be taken into account in future predictive validity studies. Implications of this effect are not yet clear, but given the considerable changes underway in selection processes (Hughes 2002; Elliott & Epstein 2005; Story & Mercer 2005; Powis 2008; Wilkinson et al. 2008) and medical curricula (Mercer 2009) more work needs to be done in this area.

### The interview score

At UWA, we have for over a decade delivered a highly structured interview, with on-going evaluation of its inter- and intra-rater reliability, as well as the several criteria it addresses each year (Mercer 2009). Furthermore, internal consistency as measured by Cronbach's alpha has been at least 0.85 in each year of delivery of the interview (Mercer 2009). Therefore it was pleasing to see that in this study, the interview total score proved an independent predictor of the Year level WAM for the clinical years (Years 4-6). Much of this was dictated by stronger relationships with academic performance linked to the ‘clinically’ based units rather than the ‘knowledge'-based units of the course. Similar observations have been made by previous researchers (Tutton 1997; Hughes 2002). This relationship with the WAM for the clinical years is important given that these composite scores for the overall performance each year are composed of both ‘knowledge'-based and ‘clinically’ based units. Further investigations of the contribution of the interview score to individual units and Year level WAM showed that the amount of variance accounted for was small (at most 3%) but it was consistent and in a context where all the predictor variables together accounted for a total in the range 10-20% (approximately). As Powis (2008) notes, medicine is accustomed to important small effects.

Of particular interest, the outcomes of further analyses that used only the score for *communication skills* in place of the total interview score yielded virtually identical results. This suggests that a global rating for communication skills is as useful a predictor as the total interview score itself, and that one of the main functional outcomes of a structured interview may be to assess these skills in the face-to-face setting. The use of Multiple Mini Interviews has become popular amongst the graduate entry medical schools in Australia (Harris & Owen 2007) and this format is being accepted and evaluated as a viable alternative to the traditional interview (Kumar et al. 2009). However, the results of this study suggest that the particular highly structured interview used at UWA is a suitable selection instrument for school-leavers.

### The three sections of UMAT

The stated purpose of UMAT is to identify candidates with cognitive skills and abilities which may be suitable to the study and practice of medicine (Mercer & Chiavaroli 2006). The psychometric properties of the test are monitored by the developers, ACER, and results are reported each year in a written report to the UMAT Consortium, detailing item analyses, reliability indices and candidate performance (ACER 2010).

The results from each of the three sections of UMAT did not show any significant relationship to the WAM in the regression analyses. However, there was a correlation of UMAT_1 (logical reasoning and problem solving) with the Year 5 WAM, significant at the 5% level; and there were generally weak positive associations of UMAT_1 with marks achieved in some individual ‘knowledge'-based and ‘clinically’ based units. In contrast, UMAT_3 (non-verbal reasoning) showed weak positive and negative associations with some results. The predictive validity of aptitude tests, such as UMAT, in relation to medical course outcomes clearly needs longer term assessment and evaluation and this has been acknowledged in other contexts (Nicholson 2005; Lynch et al. 2009). A careful analysis has been conducted on the construct and content validity of the UMAT (Mercer & Chiavaroli 2006). However, future work now needs to determine whether there is significant and worthwhile predictive validity of such tests in relation to both undergraduate and ultimately graduate clinical performance and the particular domains of knowledge assessed in MBBS courses and beyond.

### Analysis of the non-standard data

The 249 non-standard students who entered with a GPA from their previous tertiary studies and whose data were analysed in the same way, showed similarities and differences with the 1174 standard entrants reported here in detail. Previous academic achievement (GPA) was a consistent and diminishing predictor over the 6 years. The other consistent predictor for Years 1-3 was UMAT_2 (understanding people). Female sex was significant for Years 4 and 5 and the interview score for Year 5. Hence outcomes were less consistent, which may in part have been due to much less data being available, particularly in the latter years of the course. The outcome of note here was the influence of UMAT_2 in the first 3 years.

## Conclusion

The last 10 years have seen a proliferation of selection processes into medical courses involving an interview and the use of aptitude tests. This process, which is not entirely reliant on academic achievement, has not been without criticism (Watson 2006). It is therefore important for studies to investigate the outcomes of selection into such high-stakes courses and to assess the consequences of taking a broader approach to student selection. The finding that academic achievement was an important predictor of performance throughout our MBBS course was not an unexpected result (Ferguson et al. 2002; McManus et al. 2005). The results with respect to previous academic achievement and female sex have been reported before, so this study confirmed their place in predicting outcomes in medical courses. The results for UMAT in this study were mixed and relatively weak, except for the non-standard entrants for whom UMAT_2 (understanding people) was effective across Years 1-3. However, given the strong construct and content validity of the test (Mercer & Chiavaroli 2006) and the potential for association with clinical reasoning skills, evaluation of the utility of this test and other such aptitude tests has only just started and may need to extend beyond medical school.

The outcomes of the interview formed the aspect of most interest in this study. Conducting interviews is a resource-intensive undertaking for medical faculties (Hughes 2002; Powis 2008) and the use of such resources, both financial and personnel-based, has been questioned (Norman 2004). The nature of the interview delivered at UWA makes it particularly expensive in human resources. Given that the effects of the interview were predominant in the latter years of the course and especially in ‘clinically’ based units, it is possible that the predictive value of a selection interview may well become even more apparent during clinical interaction after graduation (Peskun et al. 2007). Furthermore, it seems that the graduates themselves value good communication skills as a method of furthering their careers (Hyde et al. 2010). It therefore seems logical that selecting candidates with the potential for communicating effectively with peers and patients, and then continuing to develop this skill during their course, should fit them well for their career. The utilisation in the selection process of an assessment of communication skills, through a structured interview, is supported by the results of this study.

This study has confirmed expectations with respect to previous academic achievement and raised the issue of the sex effect. However for the purposes of the Faculty of Medicine, Dentistry and Health Sciences at UWA, it is a positive step towards validating the use of the structured interview with an emphasis on communication skills. Evaluation and validation of all selection criteria should be an on-going priority for medical schools, including the period after graduation.
